# Supplementary material for: Are carbon emissions trading and green financial instruments synergistic? -Comprehensive quantitative research based on content analysis
Source: PLoS One. 2024 Mar 7;19(3):e0298601. doi: 10.1371/journal.pone.0298601 (PMC10919601; doi:10.1371/journal.pone.0298601)
Supplement: S1 Data — (ZIP) [file pone.0298601.s001.zip › Data/地级市Z_F工作报告.zip!/╔╜╢1⁄2/╝├─■╩╨/└┤╫╘╣1⁄2╓┌║┼í╛┬φ┐╦╩2╛▌═°í┐_2009.docx]

各位代表：

　　现在，我代表济宁市人民政府向大会报告工作，请予审议，并请市政协委员和其他列席会议的同志提出意见。

2008年工作回顾

　　刚刚过去的一年极不寻常，极不平凡。在中共济宁市委的坚强领导下，全市上下坚持以科学发展观为统领，全面贯彻党的十七大和十七届三中全会精神，认真执行国家、省加强改善宏观调控一系列决策部署，以 “学苏北精神，促济宁跨越”活动为动力，不断解放思想，锐意开拓创新，勇于攻坚克难，凝心聚力实干，全市经济社会实现又好又快发展，新一届政府各项工作开局良好。

　　（一）紧抓第一要务，保持了经济平稳较快发展。初步预计，全市地区生产总值突破2000亿元、增长13%以上，地方财政收入119.45亿元、增长18%，规模以上固定资产投资796.8亿元、增长25.2%。粮食单产、总产均创历史新高。各类规模种养基地发展到2760处，新增无公害、绿色、有机农产品认证121个，市级以上重点龙头企业发展到255家。防治灰飞虱、美国白蛾等动植物疫病取得重大胜利。全市发放支农政策性补贴9.5亿元，市县财政拿出2000多万元专项资金扶持奶业发展。渔业品牌建设得到加强。林业产业化步伐加快。农业综合机械化水平提高到67.2%。泗河等骨干河道治理大见成效，完成65座大中小水库除险加固任务。规模以上工业企业发展到3213家、比上年增加651家，主营业务收入3090亿元、利税410亿元、利润268亿元，分别增长36%、32%和37%。煤电化工、机械制造、生物技术、纺织服装、食品医药五大产业集群增加值占比达到40.1%。太阳纸业销售收入突破百亿元。高新技术产业产值超过千亿元、占比提高到31%。济宁高新区主导产业加速成长、综合实力显著增强。14个省级经济开发区投资强度加大、项目密度增加。制定落实扶持中小企业发展政策措施，民营经济贡献份额稳定提高。全社会消费品零售总额729亿元、增长23.3%。一批重点物流企业快速膨胀。孔子文化品牌带动战略深入实施，区域文化产业带和重点园区建设步伐加快，曲阜新区文化产业园跻身国家级文化产业示范园区。汶上宝相寺、微山南阳古镇、泗水西侯幽谷等景区开发建设初具规模。推行市内旅游“一卡通”。全年旅游社会收入149.6亿元、增长19.5%。工商、物价执法管理措施加强，食品药品质量监管体系逐步完善。借助中国专利高新技术产品博览会及“院士济宁行”活动平台，引进新技术、新成果860项和急需人才840名。新建5个省级以上工程技术中心和23处产学研基地，全年取得重要科技成果175项，专利授权2650项。开工建设国家煤及煤化工产品质量监测检验中心。节能减排专项整治力度加大，列入国家、省治污规划重点项目竣工率达到95%以上，淘汰87家企业落后产能。治理采煤塌陷地1.5万亩。修复新建人工湿地3.85万亩。

　　（二）抢抓政策机遇，实现了投资规模持续扩大。国家、省实施扩大内需一系列政策举措后，随即成立市扩大内需工作领导小组，制定出台17条具体贯彻落实意见，及时筛选一批重点项目向上对接提报，先期争取到1.98亿元专项资金，102个项目进入国家、省“大盘子”。持续开展境内外系列招商活动，全年实际到位市外国内资金313亿元、增长36%；新批办外资项目50个，直接利用外资3.8亿美元、增长30%；完成进出口总额34.3亿美元，其中出口19亿美元、增长10%。举办大规模银企对接活动，金融机构年末存贷款余额分别达到1460.3亿元和864.6亿元。全年新开工规模以上项目3172个，其中亿元以上项目91个，吉利汽车零部件、小松工业园等一批高大外项目加紧建设，森达美系列项目顺利推进，21个亿元以上产业大项目竣工投产。

　　（三）锐意改革创新，办成了一些关系全局和长远的大事实事。顺利调整市中区、任城区行政区划，新设立北湖旅游度假区。14个镇率先实施新型乡镇建设试点，“镇园合一”管理体制开始运转。理顺市县财政收入分配体制，开征价格调节基金，推行国有资本经营预算制度，基本完成驻济外地煤矿企业属地注册纳税。新设立建设、交通、水利和北湖度假区四个投融资公司，重大基础设施建设多元融资格局初步形成。以济矿为龙头组建济宁能源发展集团。促成如意集团重组樱花纺织。引入山东银座盘活济宁华联。强力推进市属工业困难企业改制脱困，35家企业依法破产终结。国有及控股企业法人治理结构逐步健全。争取到来往京沪动车组在兖州停靠。事业单位改革稳步推进。市级机关后勤管理体制改革取得新进展。招投标监管机制更加完善。市县行政审批中心服务效能不断提高。统计调查体制调整到位。

　　（四）主攻重点工程，提升了城市品位形象和服务功能。完成《济宁市城市总体规划》修编任务。提速推进中心城区开发建设，启动总投资80亿元的47项重点工程，完成投资40亿元。济宁曲阜机场投入运行。孔子文化会展中心、圣都国际会议中心落成启用。基本建成微山二线船闸和济宁港跃进港区。一批国省道及城区主要道路延伸拓展和崇文大道升级改造、济邹路洸府河大桥改建工程竣工。太白楼路东西跨河大桥、运河路高架桥和北湖中路、济安桥路南延工程加紧推进。人民公园、百花公园改造和杨桥绿地、滨河体育公园及全民健身广场一期建成开放。引水入城工程提前完成。新增公共绿地548万平方米。中心城区完成拆迁面积245万平方米。济北新区、王母阁片区和三郭片区等重点片区开发相继启动，对25个城中村实施联片改造。及时出台促进房地产业健康发展的扶持政策。县域城市改造建设力度加大、品位提高。重点小城镇建设初具规模。行政综合执法能力切实加强。治理超限超载车辆专项行动成效明显。秸秆禁烧长效机制基本形成，异味污染得到遏制，城区空气质量优良率保持在90%以上。

　　（五）高度关注民生，改善了城乡群众生活质量。市级财政用于直接关系民生支出8.2亿元、增长48%。年初确定的为民所办10件实事全面完成。全市新增城镇就业8.6万人，城镇登记失业率3.94%。城镇居民人均可支配收入15670元，农民人均纯收入5928元，分别增长12.8%和12.5%。新增养老、失业、工伤保险参保人员12万人。再次提高企业退休人员养老金、城乡低保补助标准。调高新农合筹资标准和报销比例，农民参合率提高到97.46%。城镇居民医疗保险试点全面推开，69.8万人纳入保障网络。创建“无障碍市”和实施“千人助行”、“千人助听”系列助残济困工程成效明显。新建农村公路1260公里、改建危桥86座。投资822万元为50个乡镇敬老院安装取暖设施。全面建立廉租住房制度，建设经济适用房39万平方米。资助2415户贫困农民改建住房。城区15.6万户居民用上清洁安全的天然气，集中供热面积突破900万平方米。全部免除城乡义务教育学杂费。资助贫困家庭学生16万人。改造中小学校舍危房25万平方米。山东贸易职工大学新校区一期工程竣工，济宁技术学院完成扩容整合。济宁学院办学水平稳步提高。成功举办国际孔子文化节、首届世界儒学大会和省第二届文博会。全运会赛事场馆建设进度加快，群众性体育活动丰富多彩，奥运火炬曲阜站传递圆满成功。基本完成乡镇卫生院改造任务，建起1788个规范化村卫生室，城市社区卫生服务人口覆盖率达到90%以上。人口自然增长率控制在6‰以内。加强社会治安综合治理，深入开展严打整治斗争，人民群众安全感不断增强。司法行政基层网络进一步健全。群众工作平台服务功能更加完善，领导干部大接访活动成效明显。国家安全工作扎实深入。安全生产主要控制指标连续6年呈下降趋势。社会应急管理得到加强，及时妥善处置了问题奶粉等突发公共事件。荣获省级文明城市称号。国防动员教育、民兵预备役建设和双拥共建富有实效。新闻出版、广播影视、档案史志、民族宗教、外事侨务、人防地震、水文气象、妇女儿童、老龄、慈善、红十字等项事业取得新成绩。

　　各位代表！“5.12”四川汶川特大地震牵动了济宁人民的心。全市累计捐款捐物2.82亿元，调运去大批救灾急需物资，先后组织近千人专业队伍深入灾区展开救援工作，帮助恢复重建，赢得了当地党委、政府和干部群众的广泛赞誉，孔孟之乡扶危济困、乐善好施的传统美德在抗震救灾斗争中焕发出新的时代光彩！

　　（六）坚持依法行政，增强了政府执行力公信力。扎实推进法治政府建设，清理取消行政审批事项459项，进一步规范了行政处罚裁量权。全年办理人大代表建议183件、政协提案419件。坚持重大问题调研论证、专家咨询、公示听证等科学民主决策制度。向社会公开市政府组成人员工作职责、重点任务及服务承诺。电子政务专网平台安全高效运转。新闻发布制度日益完善。“市长公开电话”及时听取解决了群众反映的大量实际问题。细化分解责任目标，“三重”工作高质量、高效率推进。出台强化行政问责、效能监察的6个规范性文件，严肃查处一批不作为、乱作为的违纪违规案件。认真执行中央、省廉政纪律和《济宁市国家工作人员十个不准》，全面落实党风廉政建设责任制。强化经济责任审计，持续开展治理商业贿赂和纠正部门行业不正之风工作，严惩了少数腐败分子。

　　各位代表！面对异常复杂的环境，异常严峻的挑战，异常艰巨的任务，我们取得这些成就实属不易。这里，我代表济宁市人民政府，向全市工人、农民、知识分子和干部，向人大代表、政协委员和各民主党派、工商联、人民团体和各界人士，向中国人民解放军驻济部队指战员、武警官兵和公安干警，向中央、省驻济单位，向支持参与济宁建设发展的港澳台同胞、海外侨胞和国际友人，表示衷心感谢和崇高敬意！

　　实践充分证明，市委审时度势、科学务实，各项决策是完全正确的；各级各部门恪尽职守、无私奉献，是能打硬仗的；广大干部群众勤劳智慧、奋发有为，是富有创业激情和创造活力的！实践也使我们深切体会到，只要我们上下同心、团结一致，就没有克服不了的困难；只要我们坚持科学发展、跨越发展，就没有实现不了的目标；只要我们直面挑战、共克时艰，就没有创造不了的奇迹！我们可以自豪地说，别人能办成的事情咱们济宁人也能够办成，别人办不成的事情咱们济宁人照样能够办成！

　　我们也清醒地看到，全市经济社会发展仍存在一些差距和不足。受国际金融危机影响，去年三季度以来经济增速明显放缓，工业效益出现下滑，企业亏损面、亏损额增加；外贸出口严重受挫，利用外资规模偏小；主导产业竞争优势不突出，发展方式没有根本转变；农业结构调整深度不够，农民持续增收难度加大；就业社保和社会稳定面临新的压力，完成节能减排约束性指标任务艰巨；政府职能转变不够到位，少数部门和工作人员作风不实、效率不高、服务不优，影响发展环境的问题时有发生。对此我们一定高度重视，切实找准症结，采取得力措施，认真加以解决。

今年经济社会发展主要任务

　　2009年是新中国成立60周年，是深入实施“十一五”规划的关键一年，也是推进我市科学发展、跨越发展，打造区域发展新高地的重要一年。做好新一年政府工作，我们面临着严峻的宏观经济形势。国际金融危机仍在扩散蔓延，全球增长减速与国内周期性调整叠加在一起，影响经济发展的不确定因素和潜在风险还在增加。我市经济出现的短期问题与长期积累的深层次矛盾相互交织，下行压力持续加大。各地在应对挑战中纷纷调整经济结构，力图把握主动、抢占先机，区域竞争日趋激烈。复杂多变的环境既带来空前困难，也蕴含重大机遇。在积极的财政政策和适度宽松的货币政策大背景下，国家、省保增长扩内需调结构一系列政策措施深入落实，对整个经济的激活拉动效应正在逐步显现；省委、省政府持续加大对鲁南经济带开发建设的扶持力度，一批重大基础设施和产业项目陆续启动，助推发展的内生动力将会迅速释放；我市经济较快增长的基础没有根本动摇，已经具备一定抵御风险的能力，一批大项目、好项目进入集中收获期，城市承载能力和服务功能显著增强，充裕的能源资源和优越的地理位置又使我们在争取上级政策扶持上占据独特优势；金融危机催生了调整经济结构、转变发展方式的倒逼机制，将使我市加快战略转型、提升产业层次的行动更加自觉、步伐更加坚实；尤其是全市上下思想解放的不断深入，机制体制的不断创新，驾驭市场经济能力的不断提高，这些都为我们推进经济社会又好又快发展提供了强力支撑。我们务必辩证认识形势，始终保持奋发有为的精神状态，坚定攻坚克难的必胜信心，形成保增长促发展的强大合力，更加敏锐地抢抓机遇，更加科学地谋划发展，更加有效地破解难题，努力夺取全市科学发展、跨越发展的新胜利！

　　今年工作的总体要求是：全面贯彻党的十七大和十七届三中全会精神，深入落实科学发展观，围绕打造区域发展新高地的战略目标，以保持经济平稳较快发展为首要任务，提振信心，应对挑战，积极作为，科学务实，坚持“科学发展跨越发展”总基调，坚持项目建设、招商引资、园区发展“三项重点工作格局”不动摇，突出投资拉动、消费促动、创新驱动，保增长、保民生、保稳定，促就业、促转型、促开放，加快政府职能转变和管理创新，奋力开创经济社会又好又快发展新局面。主要预期目标为：地区生产总值增长12%，地方财政收入增长9%，全社会固定资产投资增长22%，社会消费品零售总额增长16%，外贸进出口增长12%，引进市外国内资金增长10%，直接利用外资增长20%，城镇居民人均可支配收入和农民人均纯收入均增长8%，城镇登记失业率控制在4.5%以内；万元地区生产总值能耗降低5.57%，二氧化硫排放减少2%，化学需氧量排放降低5%，人口自然增长率控制在6.5‰以内。重点抓好八个方面的工作。

　　（一）集中突破重大项目，增强投资对经济增长的拉动力。紧紧抓住国家扩大内需的难得机遇，准确把握相关政策导向和投资重点，围绕推进重大基础设施、公共设施、先进制造业、现代服务业、高新技术产业、新农村建设及保障民生、节能环保等领域的重点工程项目，多方融通资金，持续加大投入，加快建设步伐，为当前和更长时期经济跨越发展注入活力、积蓄后劲。

　　抓基础设施建设增加有效投入。开工晋煤东运专用线梁山至兖州段。配合搞好新菏、兖石和京九铁路电气化改造，加紧实施京沪高铁济宁段及曲阜站点建设。加快推进济徐高速公路济宁段工程。争取微山湖跨湖高速尽快立项。及早兴建机场及港口疏港道路和滨湖路二期。抓好洙水河航道、济宁至东平湖三级航道改扩建和内河港口群建设。推进南四湖湖内行洪、湖西大堤续建、治淮东调南下、泗河治理和南水北调东线济宁段系列工程。启动新一轮城乡电网改造建设。及时落实国家、省投资项目配套资金，强化资金使用和工程质量监管。继续筛选一批国家支持类项目向上提报，争取更多项目进入国家、省扶持计划。

　　抓招商引资增加有效投入。顺应国内外产业资本转移的新趋势，持续展开招商攻势。突出抓好资源招商，督促现有资源型企业积极谋求与大公司、大集团及战略投资者的合资合作，兴办资源精深加工、接续替代产业和高新技术项目。突出抓好产业链招商，围绕培植主导产业集群和建设千亿元大产业，集中在煤电化工、工程机械、汽车及零部件等领域引进一批基地型、龙头型大项目，兴办更多上下游配套协作项目。突出抓好园区招商，发挥“镇园合一”体制优势，强化招商工作团和专业招商队伍责任，面向重点国家地区实施整体推介、常年组织招商，以招商助推开发建设、加速项目集聚。突出抓好企业招商，鼓励各类企业拿出优势资产实施嫁接改造，支持骨干企业与跨国公司、世界500强的强强联合。突出抓好公共设施招商，争取在城市供水、污水处理、垃圾处理及大型场馆运营和教育、医疗机构建设等领域率先突破。创新运作模式，凸显国际孔子文化节等系列节庆招商活动成效。探索私募基金招商、跨国并购招商新模式。严格依据国家产业政策和相关法规取舍项目，杜绝新上高耗能、重污染项目。

　　抓银企合作创新增加有效投入。健全完善银政银企合作机制，鼓励金融机构扩大信贷规模、支持地方经济发展，加强与市外知名商业银行联系协作，定期举办银企对接及贷款项目推介活动，力促信用总量持续扩充。支持商业银行深化内部改革、改善金融服务。推动城市商业银行增资扩股，鼓励农村信用社壮大实力，积极发展邮政银行，引进国内外金融企业来我市设立分支机构。优化金融生态环境，提升企业和全社会信用水平。

　　（二）推进农村改革发展，力促农业增效农民增收。认真贯彻党的十七届三中全会《决定》和省、市委全会决策部署，按照统筹城乡发展的要求，着力创新“三农”工作思路，落实强农惠农政策，解决突出矛盾问题，巩固发展农业农村经济好形势。

　　加大投入强化农业基础。健全农业投入保障机制，市级财政安排“三农”支出5.2亿元，比去年增长10%。今冬明春实施1.36万项农田水利建设项目，完成62座小水库除险加固任务。抓好“农村水网”示范区建设。推行“村村通自来水”工程市场运营模式，完成3.5万农户“一池三改”任务。改造农村公路800公里、桥梁50座。加快推进农业生产全程机械化，推广农作物秸秆综合利用新技术。办好各类农业科技示范园，充实加强“科技特派员”队伍，依托农村远程教育网络提供市场供求信息、普及应用良种良法。健全市县乡三级农产品质量监控检测体系，强化农资质量监管，超前防范、有效应对各种动植物疫病灾害。

　　调整结构壮大特色产业。稳定粮食面积，加快国家优质粮食产业工程和国家新增大型商品粮基地建设。培植一批高产稳产地块和吨粮县乡，切实保障粮食安全。扩张优质、高效、生态、安全农产品生产规模，放大农产品质量安全区示范带动效应，新创一批无公害、绿色、有机农产品品牌。结合实施土地流转，支持有条件的地方发展优质粮棉油、精细蔬菜瓜果、畜牧水产、园艺花卉等特色产业产品适度规模经营。因地制宜发展设施农业、城郊型农业、观光休闲农业。实施龙头企业培植“333”工程，推进益海嘉里粮油等农产品精深加工项目，年内新增规模以上龙头企业50家。依托规模种养基地创办多种形式的合作经济组织。改造提升一批重点农产品产地市场。支持各级供销社面向农村建设现代流通服务网络。畅通“绿色通道”，降低农产品运销成本。

　　拓宽渠道促进农民增收。充分挖掘农业内部增收潜力，鼓励农民发展多种经营、创办“短平快”增收项目，通过股份合作参与兴办农业资源开发及二三产业项目。坚持就地转移、异地输出与自主创业一起抓，分门别类搞好技能培训和配套服务，内外并举开发转移就业基地，落实返乡农民创业扶持政策，最大限度开辟农民工就业岗位，力争全年农村劳动力转移就业规模不低于上年水平。建立严格规范的直补渠道，确保各类农业补贴资金直接发放到农民手中。有效控制农资价格，继续清理涉农收费，千方百计把农民负担降到最低限度！

　　创新制度推进农村改革。深化新型乡镇建设试点工作，进一步理顺管理体制，明确职责权限，镇园一体整合资源、科学规划，协调推进产业发展、社区建设与公共服务。稳定完善农村土地承包关系，坚持依法、自愿、有偿的原则，有序推进土地流转。探索鼓励农民“离土进城”的有效政策，促使在城镇稳定就业和居住的农民逐步转为城镇居民。积极稳妥实施集体林权制度改革。争取创建镇村银行，增加小额贷款公司试点，鼓励兴办农村资金互助合作组织。扩大农业保险覆盖范围。

　　（三）强化扶持保障措施，加速振兴工业经济。分类指导加大企业帮扶服务力度，调整结构加快推进产业升级，促使工业经济壮大实力、提速增效。

　　引导企业练好内功。完善工业经济运行监测预警机制，强化重点行业、重点企业调度分析，开展“向企业送服务”活动，落实要素供应保障措施，确保有市场、有效益企业满负荷生产。鼓励企业利用增值税转型、进口设备免税等优惠政策，大上技术改造项目，力争今年企业技改投入占到工业投入的60%以上。帮助企业健全营销网络，全力开拓市场，突出抓好原材料类、装备类地产品的直供配送，提高重点工程市内产品配套率。落实出口退税政策，强化海关、检验检疫等部门协调配合，支持企业调整进出口商品结构，开辟工业品出口新兴市场，优先进口急需技术装备和重要原材料。鼓励有条件的企业到境外投资开发战略资源、创办加工制造业项目。引导企业加强内部管理，最大限度地消化减利因素。督促企业开展全员培训，培养高素质企业家队伍。

　　促进工业结构优化升级。落实主导产业特别是四大千亿元产业结构调整年度目标，围绕能源工业、煤化工、装备制造、食品工业产业链条延伸、优势产能扩张及名优品牌培育，加快推进国宏100万吨甲醇、卡博特碳黑、峄化DMF、华润微山湖电厂、中油燃气二甲醚、恒立醇氨、小松精密铸造、山推重工科技园、重汽二期、玉柴发动机和福林汽车零部件等一批行业龙头项目，力争两年内建成3个过500亿元大产业，机械制造业率先突破千亿元。抓好光通信、风能发电变流器、电子级玻璃纤维等一批高新技术产品开发与扩能。集中在机电一体化、电子信息、新能源、新材料和节能环保等领域开展科技招商，引进一批高新技术产业化项目。加紧运作中钢多晶硅、新力精密机械、鲁抗辰欣、新风光节能产业园等高端大项目。力争济宁高新区晋升国家级高新技术产业开发区。年内新增国家级高新技术企业30家，高新技术产业产值占比提高3个百分点。

　　积极培育大企业大集团。实施“骨干企业梯次发展计划”，支持重点工业企业吸纳优势资源，迅速膨胀规模。鼓励行业龙头企业兼并落后企业、困难企业，推进关联产业、上下游企业融合重组，支持太阳纸业集团实施销售收入三年过200亿计划，年内争取如意集团、银河集团销售收入过百亿，3家企业过50亿，2家过30亿。抓好骨干企业规范化改制，充实上市后备企业队伍，支持有条件的企业尽快上市。加强国有及控股企业监督管理，建立外派监事会制度。加快济能发集团整合重组和上市步伐。继续抓好困难企业改制脱困，确保列入计划的企业尽快完成破产终结。

　　增强中小企业发展活力。扎实推进“中小企业成长计划”，指导帮助企业改进工艺装备，实施挂强联名，搞好科技创新，优化产品结构，努力提高抵御市场风险的能力。完善信用担保体系，提升各类担保机构资质水平，广泛推行联保融资模式，设立中小企业偿贷周转金，有效破解融资瓶颈。持续兴起全民创业热潮，降低创业准入门槛，鼓励各类能人和有创业欲望的人员兴办民营项目，不断增加规模以上企业数量。年内全市民营经济增加值、上缴税金均增长20%以上。

　　（四）努力激活消费需求，繁荣发展服务业。完善政策体系和专项规划，增加引导资金投入，运用现代理念、现代方式和现代技术，推动服务业加快发展，年内力争服务业增加值占地区生产总值、服务业投资占全社会固定资产投资、服务业税收占地税收入“三个比重”提高1-2个百分点。

　　提升文化旅游产业综合带动效益。大力实施孔子文化品牌带动战略，有机整合文化资源，布局建设各具特色的文化产业聚集区，支持曲阜新区国家级文化产业示范园区加速崛起。抓好特色文化产品系列开发，积极引进动漫、广告、影视制作、形象设计等创意文化项目。探索报业、出版、广电、演艺集团化发展、产业化经营路子。编制济宁城区文化旅游产业发展专项规划，先期实施古运河文化旅游景观带系列开发项目。加快推进曲阜明故城、邹城“四孟”、汶上宝相寺、泗水西侯幽谷和万紫园、微山湖旅游区、梁山景区等旅游资源深度开发，抓好太阳国际、香格里拉等星级酒店及旅游服务设施配套建设。持续增发“一卡通”，倡导“济宁人游济宁”活动。抓住济宁曲阜机场通航有利时机，尽快实现我市与国内重点旅游城市的市场对接、客源共享。年内全市接待中外游客突破2200万人次，旅游社会收入突破160亿元。

　　促进房地产业持续健康发展。认真落实上级和我市出台的各项扶持政策，最大限度减少有关规费征收，进一步降低房地产开发与消费成本。协调金融机构增加房地产信贷规模。严格执行基准地价和建设用地“招拍挂”制度。引导开发企业调整优化供房结构，增加小户型普通商品房投放量。放宽二套房政策，搞活住房二级市场。整顿规范房地产开发市场秩序，督促市内开发企业强化管理、改善装备、提升资质、增强竞争实力。

　　搞活商贸流通业。放大知名商业品牌带动效应，有计划推进一批国有商贸企业改制重组。采取中心城区辐射、城镇乡村联动的方式，兴办更多连锁、超市等新兴流通业态项目。继续实施“万村千乡”市场工程，扩大家电下乡补贴政策覆盖面。积极培育汽车、健身、休闲等消费热点，促进消费结构优化升级。

　　大力发展生产性服务业。加快推进森达美战略合作系列项目，尽早建成龙拱河等重点港口，同步完善仓储服务区等配套设施。规划建设一批大型物流中心，集中扶持新华、交运等重点物流集团，支持骨干企业发展第三方物流。依托现有场馆扶持发展会展业。积极培育资本市场。鼓励重点园区兴办软件产业，发展服务外包业务。

　　（五）着力转变发展方式，注重抓好自主创新和节能减排。把推进自主创新作为转变发展方式的中心环节，深入实施科教兴市和人才强市战略，加快建设创新型城市。一是强化企业自主创新主体地位。着力促进创新政策向企业倾斜、创新成果向企业集中、创新人才向企业流动，督促各类企业提足用好技术开发经费，年内再创建5个省级以上工程技术中心，扶持发展150家民营科技企业。二是巩固发展产学研合作创新联盟。鼓励企业与高等院校、科研院所开展多种形式的联合协作，建设鲁南工程技术研究院，提升鲁南质检中心研发服务功能，培育10个市级产学研合作创新工作站，发展100个产学研合作创新基地。三是主攻引进消化吸收再创新。集中研发经费用于创新成果产业化重点项目，带动更多社会资金投向“二次创新”。引导各类企业吸收转化最新技术工艺。整合市内企业及驻济高校、科研院所研发力量，围绕主导产业培植和节能降耗、污染防治，开展共性、关键、核心技术联合攻关，开发一批拥有自主知识产权的新产品。四是建设创新人才高地。发挥驻济高校和职业技术院校优势，为重点行业、骨干企业定向培养技能型人才。继续实施“千名人才引进计划”，舍得花本钱面向海内外引进高层次急需人才。鼓励支持学科带头人、专利持有人及研发创新团队来我市创业发展。重视引进国外智力。积极发展创业风险投资。重奖创新创业贡献突出的优秀人才。

　　坚持节约优先、环保优先，确保完成节能减排年度控制目标。一是加快推进重点治理项目。分解落实6个重点行业、100家重点企业和省级经济开发区节能减排年度目标，实施100个节能改造项目，强化19家废水排放企业点源治理，加快建设7处截污导流工程，完成12家城市污水处理厂管网配套建设，及早兴建济北新区、北湖度假区两座污水处理厂。巩固扩大燃煤电厂脱硫改造治理成果。积极推进化工企业“退城进园”。完成年度落后产能淘汰任务。严禁新上“十五土小”项目。二是大力发展循环经济。全面推行企业环境质量管理认证，完成30家企业能源审计、清洁生产审核，抓好10个“三废”资源化项目，集中培植30家循环经济先导示范企业，选择部分园区和企业探索推行原料产品可循环利用、废弃污染物零排放的绿色产业集群发展模式。三是强化执法监管。落实差别电价、超标准耗能加价等政策。严格排污总量控制和排污许可证管理。认真执行新上项目“三同时”和“一票否决”制。尽快实现重点排污企业和主要河流断面在线监测“全覆盖”。定期开展环保执法专项行动，严厉查处违法排污行为。四是抓好集约节约用地和生态保护重建。全面完成市县乡三级土地利用总体规划修编。多方挖潜盘活存量土地，鼓励发展高层厂房，对重大项目用地实行“点供”，杜绝违法用地行为。推进环南四湖大生态带和九龙山区生态修复建设工程。修复新建南四湖人工湿地4.8万亩。有效控制农业面源污染。完成1万亩采煤塌陷地整治任务。加快荒山、荒滩造林绿化，年内森林覆盖率提高到26.5%。

　　（六）扩张开发建设规模，进一步拉开城市布局框架。把推进城市化作为保增长促发展的重要着力点，依据新一轮城市总体规划，争取用好政策，拓展融资渠道，以基础设施、重大工程为牵动，实施重点区域和关键部位的饱和投入、集中突破，力求建设规模更大，推进速度更快，城市面貌变化更显著。

　　高水准编制城乡规划。聘请高层次设计力量，及早完成重点片区、地段控制性详细规划和一批专项规划，做好年度重点工程项目修建性规划。指导督促县乡统筹抓好镇村规划修编。加紧设计兴建市规划展示馆。强化规划执法刚性，严查违法建设行为。

　　高质量建设重点工程。突出抓好太白楼路东西跨河大桥、运河路高架桥、济安桥路公铁立交和文体中心、金融中心、全民健身广场二期等16个续建项目。尽快开工市区二环线及滨湖生态景观工程。启动市区车站南路、北湖中路、金宇路、荷花路等主干道延伸改建工程。及早兴建城区雨污分流工程。加紧实施华能济宁电厂、东郊热电厂扩容及供热管网拓展。规划建设南池公园、生态湿地公园和西郊公园。抓好6条城区道路沿线绿化。

　　高效率推进片区开发。依据规划科学制定北湖度假区各功能分区招商开发专案，先期抓好路网框架、水系整治和公共设施配套，集中突破文化教育聚集区主体工程，适时启动旅游、商贸和住宅区建设。探索济北新区、王母阁片区系列工程项目整体推介、融资开发新模式，统筹推进行政、商务与住宅聚集区建设。以佳世客中心建设带动三郭片区整体开发，同步实现城市功能完善、新兴产业崛起、居民生活质量提高。开工一批城中村重点改造项目。引导县市区协调抓好旧城改造与新区开发，鼓励支持兖州、曲阜、邹城与嘉祥依托重点工程和经济园区，加快与中心城区对接融合。以人为本落实城市拆迁各项政策措施，严格按规定标准及时兑现补偿，保证如期安置到位。强化各类投融资平台融资能力建设，放大政府投资吸附集聚金融资本、社会资本、外来资本投入城市建设的“乘数效应”，更多地运用BT、BOT模式推进重大工程项目建设运营。争取转借中央国债，发行城市建设债券。

　　高效能强化城市管理。分层次落实城市管理主体责任，全面推行街道社区网格化管理和居民自我管理。切实强化公共设施维护管理，组织开展行政综合执法专项行动，突出抓好城市出入口、城乡结合部及公共聚集场所、背街小巷的集中整治。巩固扩大城乡一体“大公交”发展成果，有序调控城市交通，查禁各种违法运营车辆，提高道路通行能力，使广大市民出行更方便！

　　（七）坚持增收节支并重，切实提高财政保障能力。贯彻积极的财政政策，精心做好生财聚财、理财用财的文章，广开财源，厉行节约，确保财政收入稳定增长，满足各项重点支出需要。一是做大财源“蛋糕”。用好各类专项扶持资金，积极支持经济效益好、税收贡献大、地方留成多的优势企业和重点项目。及时帮助新建企业、技改项目落实要素供应，尽早竣工发挥产出效益。督促引导大中企业加快实施二、三产业分离。对土地、矿产、旅游景点、城市公共场地设施的运营，全面实行招标拍卖制度，充分挖掘资源性非税收入潜力。二是严格税收征管。针对行政区划和财政体制调整带来的新情况，严密落实税收征管措施，对重点税种、重点行业、重点企业实行重点监控、直接调度，对市内重大基础设施及重点工程建设形成的税收实行跟踪征管，巩固扩大驻济煤矿属地注册纳税工作成果，继续推广普及税控机和发票“双奖”办法，促进社会综合治税网络向薄弱环节和征管盲区延伸覆盖。认真执行国家燃油税新政策。收足收好国有资本经营收益。进一步清理现行税收优惠政策，切实强化税务稽查，严厉打击偷逃骗税行为。三是保障重点支出。坚持集中财力办大事，进一步优化支出结构，优先保证工资发放、机关运转、民生改善、社会稳定等方面的支出需要。牢固树立过紧日子的思想，大力开展节约型机关和节约型社会建设，严格控制行政经费预算，严格控制一般性开支。今年公务购车和用车经费、会议费、接待费、出国经费一律以上年预算为基数缩减5%，挤出更多财力用来为人民群众办事、为城乡百姓造福！

　　（八）突出保障改善民生，全面加强社会建设。践行以人为本、执政为民的宗旨，强化社会管理和公共服务，尽心尽力解决群众最关心、最直接、最现实的问题。今年市级财政安排民生支出9.2亿元、增长12%。继续为城乡群众办好10件实事。

　　千方百计扩大就业。实行更加积极的就业政策，全年新安置城镇就业8万人。发挥重大工程项目带动吸纳就业的作用，通过发展中小企业和劳动密集型产业扩充就业容量，积极开发公益性岗位，帮助城镇失业人员和困难群体再就业。用好我市被列为全国首批创业型城市试点的机遇，更大力度扶持自主创业，增强创业带动就业倍增效应。高度重视大中专毕业生就业工作，鼓励他们到基层、农村和中小企业就业创业。督促企业经营者切实履行社会责任，最大限度避免和减少裁员。完善城乡一体就业指导服务体系，抓好下岗失业人员和返乡农民工免费技能培训。维护广大劳动者合法权益，营造和谐劳动关系，使更多有就业愿望和能力的劳动者有活干、有饭吃！

　　健全城乡社保体系。着力推进以农民工、非公有制经济组织从业人员和城镇灵活就业人员为主要对象的社会保险扩面征缴。提高企业退休人员养老金发放标准，每月人均增加143元。扩大个人缴费、集体补助、政府补贴相结合的新型农村养老保险试点范围。城市低保月补助标准提高到200至249元，农村低保年补助标准提高到1000元。有机整合福利、慈善、红十字等资源，增强社会救助实效。继续实施助残济困系列工程。管好用好大病医疗救助资金。扩大城镇居民基本医疗保险覆盖面，及早把中小学、职业院校及驻济高校学生全部纳入保障范围。新农合筹资标准提高到100元，相应调高报销比例。完善病种限价付费制度，创办更多“惠民病房”。全面实施保障性安居工程，年内建设经济适用房36万平方米，为1500户困难群众提供廉租住房保障，资助4400户农村贫困家庭新建住房，力争用2-3年的时间，基本解决城镇低收入家庭的住房问题。我们有信心有能力通过强化保障扶助，确保城乡群众正常生活水平稳定提高，困难群体基本生活不出问题！

　　协调发展社会事业。深入实施教育优先发展战略。巩固提升基础教育，优化中小学布局结构，全面实施危房改造、“一暖三改”和办学条件标准化工程，免除农村义务教育阶段学生寄宿费，高中阶段入学率力争达到88%。大力发展职业技术教育，建设13处标准化职教中心，引导职业院校明确目标定位、实现错位发展，尽快把山东贸易职工大学改建为山东工程职业学院，济宁市技术学院改建为技师学院，加快济宁职业技术学院扩建步伐。规划建设济宁医学院新校区和市特殊教育中心。完善医疗卫生服务体系。增强社区医疗机构服务功能，建成1787个规范化村卫生室。强化城乡医院对口支援，提高县级医院和乡镇卫生院医疗服务水平。重视传染病、地方病和职业病防治工作。扶持中医药事业发展。坚持不懈抓好食品药品质量安全监管。繁荣科技文化体育事业。筹备办好中国专利高新技术产品博览会和“院士济宁行”活动。提升市县生产力促进中心档次水平。扩充科技孵化器规模。实施一批省级国家级科技专项。强化知识产权保护管理。配合搞好中华文化标志城创意征集和科学论证，抓紧抓实历史文化遗产保护工作。深入实施文化信息资源共享、广播电视村村通、乡镇综合文化站、村文化大院和农家书屋等文化惠民工程，规划建设工人文化宫，启动市图书馆、博物馆新馆等公共文化设施建设前期工作，完成城区有线电视数字化整体转换。深化精神文明系列创建活动。抓好文明小康村、生态示范村建设试点。重视发展哲学社会科学。做好大运河申遗和第三次文物普查工作。注重发挥史志、档案社会服务效能。如期完成场馆建设任务，承办好11届全运会济宁赛区比赛项目。统筹解决人口问题，持续稳定低生育水平。抓好对口援建地震灾区工作。

　　维护社会和谐稳定。深入落实“平安济宁”建设各项措施，构建严密的治安防控网络，保持严打斗争高压态势。办好市县群众工作服务大厅，畅通百姓诉求表达渠道。完善矛盾纠纷排查调处机制，健全信访工作责任制，坚持领导干部公开接访制度。持续开展普法宣传教育，强化法律服务和法律援助。落实最严格的安全生产监管制度，深化重点行业、领域专项整治，坚决杜绝重特大事故发生。健全应急管理体制机制，完善各类应急预案，提高应对处置突发公共事件的能力。做好国家安全工作。加强国防动员教育、人防（民防）和民兵预备役建设。深入开展双拥共建。保障妇女儿童、老年人权益。做好民族宗教、外事侨务和对台工作。完善水文、气象、地震设施，提高防灾减灾能力。

　　各位代表！做好今年工作，完成既定目标任务，必须切实加强政府自身建设。我们要不断解放思想。深化“解放思想、改革创新、跨越发展大讨论”，积极投身学习实践科学发展观活动，更加透彻地认识发展大势和济宁市情，更加准确地把握科学发展、跨越发展的本质内涵，更加悉心研究和对接争取上级政策支持，强化机遇意识、创新意识和忧患意识、危机意识，切实做到开拓创新与科学求实的有机统一，突破重点与牵动全局的有机统一，抓紧当前与谋划长远的有机统一，努力提高驾驭复杂局面、应对困难挑战、领导科学发展的能力。我们要切实改进作风。坚持务实为本、效能为先，对重点工作逐一细化分解，层层明确责任，跟踪调度督查，确保落到实处。倡导特别能吃苦、特别能实干、特别能奉献精神，下决心精减会议文件，摆脱繁琐事务，深入改革发展前沿接触矛盾、解决问题。发扬雷厉风行、只争朝夕的精神，努力提高办事效率，优化政务服务，强化效能监察，严格行政问责，坚决兑现奖惩。我们要坚持依法行政。自觉接受人大法律监督、政协民主监督，认真办理人大代表建议和政协提案。完善科学民主决策机制，严格依据法定权限和程序行使权力、履行职责，按照上级部署稳妥推进行政管理体制改革。进一步精简行政许可与审批事项，健全行政执法责任制。深入推行政务公开，推进新闻发布工作制度化、规范化，加强电子政务建设，办好“12345”市长公开电话，及时倾听社情民意、化解矛盾问题、改进政府工作。我们要强化廉洁自律。全面落实党风廉政建设责任制，严格遵守各项廉政纪律规定，带头压减开支、厉行节约，切实强化审计监督，支持纪检监察机关查办违法违纪案件，认真纠正损害群众利益的不正之风，完善源头治腐制度规章，以为民、务实、清廉的良好形象赢得广大人民群众的信赖支持！

　　各位代表！新的一年里，重重困难和严峻挑战考验着我们，历史责任和崇高使命激励着我们。让我们在中共济宁市委的正确领导下，紧紧依靠全市人民，万众一心，迎难而上，积极作为，科学务实，坚定不移推进科学发展、跨越发展，朝着打造区域发展新高地的目标迈出更加坚实的步伐，加快建设民富市强、文明和谐新济宁，以优异成绩迎接新中国成立60周年！
